# From Foundation to Intelligence Integration: The Synergistic Associations of ICT and AI Support with Pre-Service Teachers’ TPACK Development

**DOI:** 10.3390/bs16060922

**Published:** 2026-06-04

**Authors:** Xu Liu, Jiaoyang Du, Jiacheng Wang, Huan Song

**Affiliations:** Center for Teacher Education Research, Faculty of Education, Beijing Normal University, Beijing 100875, China; dujiaoyang@mail.bnu.edu.cn (J.D.); 202321010122@mail.bnu.edu.cn (J.W.)

**Keywords:** TPACK, university ICT support, AI support in education, ICT self-efficacy, AI competency expectancy, pre-service teachers

## Abstract

Digital-intelligence transformation in education has made pre-service teachers’ Technological Pedagogical Content Knowledge (TPACK) a strategic concern in teacher preparation. Survey data from 11,818 pre-service teachers across 17 local normal universities in China were analyzed through hierarchical regression, quantile regression, and structural equation modeling to examine how perceived university ICT support and perceived AI support in education are associated with self-reported TPACK. Both forms of support showed significant direct and model-conform indirect associations with self-reported TPACK, but the quantile coefficients varied across the TPACK distribution: university ICT support showed a modestly fluctuating descriptive pattern, whereas AI support in education peaked at the median and attenuated at upper quantiles. ICT self-efficacy and AI competency expectancy each formed significant indirect pathways in the hypothesized model, although the ICT pathway was more strongly indirect and the AI pathway remained more strongly direct. Additional checks of university-level ICCs, cluster-robust standard errors, and measurement invariance across key subgroups supported the robustness and comparability of the findings. These patterns clarify how perceived ICT and AI support are differentially associated with self-reported TPACK and provide empirical grounds for more precise, human-in-the-loop support designs in teacher education.

## 1. Introduction

In the contemporary field of teacher education, Technological Pedagogical Content Knowledge (TPACK) has become a core framework for assessing teachers’ technology integration literacy. However, numerous empirical studies indicate that pre-service teachers still face deficiencies in effectively applying technology in authentic teaching contexts (Zhou et al., 2017; Xiong et al., 2020; Gromik et al., 2024; Çolak Yazıcı, 2026). On one hand, while research underscores the importance of student-centered digital applications (Eyal et al., 2023; Swai, 2025), their implementation in teacher education appears to remain somewhat superficial. Even within technologically advanced learning spaces ostensibly designed to cultivate professional digital competence (Tømte & Lazareva, 2023), instructional practices tend to default to unstructured exploration rather than deep pedagogical integration, thereby falling short of genuine student-centered learning needs. On the other hand, pre-service teachers tend to simplify educational technology into auxiliary tools for information retrieval or learning practice, seldom extending its use to serve as a cognitive medium for facilitating in-depth discussions and knowledge construction. It is precisely this habitual instrumental usage that results in a notable perceived gap: despite being proficient with everyday software technologies like social media and short videos, pre-service teachers often demonstrate a significant disparity when confronted with the pedagogical proposition of “how to use technology to promote subject understanding” (Deng & Wang, 2022; Lu et al., 2025). Consequently, how to configure systematic support to address this critical competency gap has become a central issue in global teacher education.

Since its proposal in 2006, the TPACK framework has consistently served as a guiding beacon for technology integration in teaching, emphasizing that teachers require a composite knowledge structure that integrates technology (TK), pedagogy (PK), and content knowledge (CK) (Koehler & Mishra, 2006). With the advancement of educational informatization, Information and Communications Technology (ICT) support, as the “first echelon” of digital transformation in universities, plays a significant role in providing the foundational environment for cultivating pre-service teachers’ TPACK through equipment, platforms, and training (Tondeur et al., 2017; Guo et al., 2026). Concurrently, the rapid development of Artificial Intelligence (AI), represented recently by tools like ChatGPT (OpenAI, San Francisco, CA, USA; https://chatgpt.com/, accessed on 1 April 2026), has enabled AI support in education to gradually demonstrate its potential for personalization, intelligence, and contextual adaptation, propelling the evolution of support modalities towards a “second curve” (Zawacki-Richter et al., 2019; Celik et al., 2026).

However, current research perspectives have shifted unidirectionally from ICT to AI support. Although AI-TPACK is widely examined as an emerging strand (Aldemir et al., 2025; Ning et al., 2024; Rui et al., 2025), few studies have attempted to integrate both paradigms within a unified framework for comparison and synthesis. Does traditional ICT support remain primarily confined to infrastructure provision and basic skills training, failing to adequately foster the development of pre-service teachers’ knowledge for deeply integrating technology into teaching? Can emerging AI support in education transcend traditional pathways to truly realize “teaching students in accordance with their aptitude” and guide pre-service teachers from “learning to use technology” to “learning to empower teaching with technology”? Furthermore, are there fundamental differences in the mechanisms of these two types of support, and how should they complement and synergize to jointly construct a new ecology for educator development in the intelligent era? These critical questions have become important issues urgently requiring in-depth exploration within the field. Therefore, this study situates university ICT support and AI support in education within the same context. Through empirical research, it aims to elucidate the specific pathways and mechanisms through which they are associated with the development of pre-service teachers’ TPACK, reveal the potential for synergy between the two support modalities, and provide theoretical reference and empirical evidence for constructing a TPACK cultivation system for pre-service teachers adapted to the intelligent era.

## 2. Literature Review

### 2.1. The Relationship Between University ICT Support and Pre-Service Teachers’ TPACK

Integrating ICT into teacher education is inseparable from university support. University ICT Support provided to pre-service teachers refers to the institutional backing in terms of facilities, equipment, professional training, and technical personnel guidance offered by the institutions where pre-service teachers and teacher educators are located, aimed at assisting pre-service teachers in applying technology to future teaching (Wang & Zhao, 2021). In this study, university ICT support specifically denotes pre-service teachers’ perception of the technical support related to ICT study and application provided by their higher education institutions, encompassing resources, curriculum design, and staffing.

Building on Shulman’s (1987) integration of PK and CK to propose pedagogical content knowledge (PCK), Koehler and Mishra (2006) further introduced TK to construct the TPACK framework. This framework aims to capture the essential characteristics of the knowledge teachers need to integrate technology into their teaching, while also accounting for its complex, multifaceted, and contextual nature. Consequently, TPACK should be understood not merely as a static knowledge structure, but as a set of overlapping and interacting knowledge domains. Its architecture comprises three core components: technology, content, and pedagogy. Based on the combinations of these three knowledge types, it forms a hierarchical structure encompassing the single-dimensional knowledge domains of CK, PK, and TK; the two-dimensional composite domains of PCK, TCK, and TPK; and the three-dimensional integrated domain of TPACK itself (Dong et al., 2014).

Existing research indicates that university ICT support consistently plays a crucial role in promoting the development of pre-service teachers’ TPACK (Tondeur et al., 2012; Farjon et al., 2019; Nelson et al., 2019; Bueno & Niess, 2023), primarily encompassing three categories: resource, curricular, and expert support. From the perspective of learning opportunities, resource support primarily involves resource access without constituting substantive learning opportunities (König et al., 2024); conversely, curricular support (e.g., short-term tool training) and expert support (e.g., specialized courses) involve deep learning over a sustained period (König et al., 2024). First, regarding resource support, early studies suggest that at the institutional level of teacher education, providing pre-service teachers with access to resources (hardware, software, learning materials, documentation, etc.) is a vital condition for integrating technology into their future classrooms (Tondeur et al., 2012). Further research points out that three types of ICT resources—desktop software (e.g., spreadsheets, word processors, presentation tools, etc.), emerging ICTs (e.g., cloud computing, social networks, learning management systems, and visual material design tools, etc.), and hardware equipment—significantly predict the formation of pre-service teachers’ TPACK framework (Kadıoğlu-Akbulut et al., 2023). Therefore, during teacher education, it is necessary for universities to systematically provide the aforementioned resources to enhance pre-service teachers’ understanding and application capabilities of various ICT tools, thereby supporting their full integration of technology into teaching practice.

Second, the systematic support provided by universities within ICT-related courses is significantly associated with the development of pre-service teachers’ TPACK. Research shows that the higher the level of technological support perceived by pre-service teachers in their courses, the more it facilitates the construction of their TPACK expertise. This support includes effective assistance such as being able to observe high-quality ICT-integrated practice cases and gaining experience in developing ICT-integrated lesson plans (Lachner et al., 2021). However, merely relying on standalone educational technology courses often proves insufficient, as this approach tends to detach learning from authentic instructional contexts. Kadıoğlu-Akbulut et al. (2023) contend that expanding technical knowledge alone fails to ensure professional TPACK competency; even teachers with solid technical foundations frequently struggle to translate this knowledge into effective teaching practice. Consequently, universities must move beyond isolated courses and instead systematically embed technology across all pedagogical coursework throughout teacher education programs, establishing a closed-loop system of continuous improvement (Tondeur et al., 2025).

Third, concerning expert support, as ICT courses are increasingly integrated into teacher education programs, the professional competence and practical ability of teacher educators themselves become particularly critical. The knowledge and experience they accumulate through designing and delivering methodology courses that integrate technology profoundly influence the depth of pre-service teachers’ understanding and the level of their practice regarding technology integration (Adnan et al., 2024; Mukuka & Alex, 2025). Research by Foulger et al. (2015) indicates that while teaching such courses, teacher educators’ own TPACK levels also showed significant growth. This growth, in turn, translates into more effective teaching demonstrations and guidance, thereby supporting the development of pre-service teachers’ TPACK. Concurrently, the more positive teacher educators’ evaluations of university IT personnel and student support are, the higher their TPACK levels tend to be (Nelson et al., 2019). This result highlights the important role of university IT support staff within the teacher education system. They not only provide direct technical assistance to pre-service teachers but also collaborate with teacher educators to co-design technology-enhanced lesson plans, deliver tailored technical training for specific pedagogical tasks, and provide real-time scaffolding during micro-teaching sessions, thereby effectively promoting the development of pre-service teachers’ TPACK.

Based on the promotive relationship between university ICT support and pre-service teachers’ TPACK, the following research hypothesis is proposed:

**H1.**  *University ICT support is positively associated with pre-service teachers’ TPACK.*

### 2.2. The Relationship Between AI Support in Education and Pre-Service Teachers’ TPACK

Early educational technology support primarily focused on the provision of hardware and software tools. However, the simple introduction of technology often fails to efficiently enhance teachers’ professional knowledge, and singular resource provision cannot achieve the goal of educational transformation. It is essential to stimulate the potential for technology integration through systematic teacher support mechanisms. In the ACOT (Apple Classrooms of Tomorrow) project, Sandholtz et al. (1997) found that the key factor in whether teachers could effectively integrate computer technology was whether they received systematic training and support regarding “how to use” and “how to use for teaching.” This finding marked a shift in the philosophy of educational technology support from tool provision to professional empowerment. Subsequent research further delineated the multi-level and systematic characteristics of effective technology integration support, identifying key elements influencing teacher technology integration as including professional development resources, technological and institutional support, organizational culture, and individual motivation, with these elements coupling and creating synergistic effects (Mumtaz, 2000). School-level supporting conditions, by clarifying goals, allocating resources, providing management and leadership support, and promoting both formal and informal collaboration among teachers, enhance teachers’ psychological and practical “readiness,” thereby improving their ability and willingness to integrate technology (Petko et al., 2018). Extending this trajectory into the current era of artificial intelligence, contemporary research emphasizes that effective AI support in education must be conceptualized as a holistic and resilient socio-technical ecosystem (Henriksen et al., 2025). Within this broader ecological framework, recent studies further highlight that successful AI integration in teacher professional development transcends mere tool adoption, requiring a deliberate focus on fostering teacher agency and providing personalized, data-driven support (Li et al., 2025).

Accordingly, AI support in teacher education constitutes a comprehensive support system encompassing digital resources, scaffolded training, institutional policies, and ethical cultures. This comprehensive framework is designed to empower pre-service teachers to integrate AI technologies into their future pedagogical practice with effectiveness, prudence, and innovation (Barbieri & Nguyen, 2025; Techakosit et al., 2026). Specifically, embodying these multidimensional elements, this support system manifests four core characteristics. First, systematicity and holism, emphasizing organic integration across technological provision, institutional arrangements, organizational culture, and individual development (Yu et al., 2025). Second, a developmental and process-oriented nature, emphasizing a progressive pathway from pre-integration, transition, development, and expansion to systematic integration, accompanying the formation and transfer of pre-service teachers’ competencies (Lai, 2024). Third, a practice-oriented focus concentrates on key contexts, such as curriculum design, classroom implementation, and learning assessment, to facilitate the transformation of abstract knowledge into actionable teaching capabilities (W. Hu et al., 2025). Fourth, ethical foresight, cultivating critical awareness and responsible literacy around issues such as data privacy, algorithmic bias, and human-AI relations, integrating ethics education as a normative dimension of the support system (A. Nguyen et al., 2023). In this study, AI support in education is defined as pre-service teachers’ psychological perception regarding the functional utility and accessibility of specific AI technology tools in supporting instructional and teaching activities within their university environment. This conceptual delineation strips away the generalized perception of macro-level policy systems and ethical cultures, anchoring instead on micro-level, tool-oriented practical support experiences. At the level of specific measurement items, AI support in education focuses on pre-service teachers’ subjective perceptual evaluations of core AI-enabled teaching and learning scenarios.

TPACK is a comprehensive knowledge framework for teachers to integrate three categories of knowledge—technology, content, and pedagogy—within specific teaching contexts to achieve effective instruction. It requires teachers not only to possess operational competence with technology but also to understand the dynamic relationships between technology, pedagogical strategies, and content knowledge, and to apply this understanding flexibly in actual teaching. Since the development of TPACK cannot be achieved solely through individual self-study but needs to be constructed gradually through the provision of resources, the creation of contexts, and guided practice, external educational support becomes a key condition for fostering TPACK formation. AI support in education represents a concretization of ICT support in the intelligent era, and its role in promoting pre-service teachers’ TPACK can be explained across multiple dimensions: resource provision, institutional guarantee, professional training, and the creation of learning contexts. Systematic resources and training not only help pre-service teachers build an understanding of basic AI concepts, functions, and application scenarios but also extend the role of educational technology support in fostering the formation of teachers’ TK and motivating technology use, enabling them to gradually develop confidence and operational capability with emerging technologies in AI-rich environments (Chai et al., 2010). Teacher demonstrations, curriculum embedding, and case analysis can provide visual exemplars of the integration of technology with pedagogy and technology with content, allowing pre-service teachers to deepen their understanding of TPK and TCK through observation and imitation (Hughes et al., 2016). Furthermore, project-driven and task-driven learning activities can further guide pre-service teachers to integrate technology, pedagogy, and content in authentic or simulated teaching contexts, fostering TPACK generation through iterative cycles of “design-practice-reflection” (Tsybulsky & Muchnik-Rozanov, 2023). This indicates that AI support in education is not merely a singular input of resources but a process of transforming external support into pre-service teachers’ internal cognitive and competency structures through systematic design and progressive experience.

Building on this theoretical analysis, the logic of how AI support in education acts on the development of pre-service teachers’ TPACK can be abstracted into a relational model. AI support in education is associated with the formation of pre-service teachers’ TPACK through multiple interacting pathways: First, it directly enhances pre-service teachers’ technological knowledge (TK) through technological resources and training. Second, it facilitates the construction of TPK and TCK by helping pre-service teachers understand the integration of technology with pedagogy and technology with content through teaching demonstrations and curriculum embedding. Third, it is associated with the dynamic integration of technology, pedagogy, and content through comprehensive learning tasks, which is associated with the emergence of the core TPACK. Based on this, the following research hypothesis is proposed:

**H2.**  *AI support in education is positively associated with pre-service teachers’ TPACK.*

### 2.3. The Mediating Role of ICT Self-Efficacy

Self-efficacy refers to an individual’s confidence and judgment in their ability to successfully complete specific actions and achieve expected outcomes (Bandura, 1977). The concept of ICT self-efficacy originated from computer self-efficacy theory, which emphasizes an individual’s assessment of their capability to use computers to complete work tasks (Compeau & Higgins, 1995), including beliefs about basic operations, advanced operations, and achieving teaching goals (Scherer & Siddiq, 2015). As the concept evolved, its scope gradually expanded to the broader field of ICT, focusing specifically on teachers’ perceptions and expectations of their ability to apply technology in teaching practice. Therefore, ICT self-efficacy refers to teachers’ or pre-service teachers’ beliefs in their own capability to effectively use information and communications technology (such as computers, the internet, and multimedia) to achieve teaching objectives (Moreira-Fontán et al., 2019; Wang & Zhao, 2021).

Pre-service teachers’ ICT self-efficacy relates not only to their confidence in using technology but also is directly associated with their teaching practice of technology integration, constituting a composite belief encompassing “being able to teach” and “knowing how to integrate” (Pozas et al., 2024). This belief can be cultivated through external support (e.g., collegial collaboration and structured course design), and its effects can be tangibly translated into technology integration competence (Dai, 2023; Ross et al., 2026). Existing research indicates that ICT self-efficacy is both a key mediating variable for university ICT support and a significant direct predictor of pre-service teachers’ TPACK (Wang & Zhao, 2021). On one hand, when pre-service teachers receive timely and adequate technological support, they perceive the process of technology use as more controllable and convenient, thereby strengthening their belief in their ability to successfully use technology for teaching (Hou & Shen, 2024; Ross et al., 2026). Research has found that both teachers’ intention and actual behavior regarding technology use are significantly shaped by the external support environment. Institutional ICT support, by providing opportunities, hands-on experience, and resources, positively predicts pre-service teachers’ positive ICT emotions (Ross et al., 2026; McCoy & Lynam, 2022). Conversely, a lack of practical environmental support, such as technical assistance or mentorship from ICT facilitators, tends to dampen the intention to use technology and is more likely to induce technostress and anxiety (Dong et al., 2020; Joo et al., 2016; McCoy & Lynam, 2022).

On the other hand, when pre-service teachers’ ICT self-efficacy is enhanced, they are more willing to invest mental effort to overcome obstacles in the technology integration process, thereby more effectively developing high-level integrative knowledge. Multiple empirical studies have confirmed that ICT self-efficacy is not only significantly and positively correlated with TPACK and its sub-dimensions but also serves as a key internal factor predicting pre-service teachers’ TPACK levels (López-Vargas et al., 2017; Ma et al., 2018; Zhao & Lan, 2017). While prior research has established self-efficacy as a crucial endogenous predictor of TPACK, recent empirical models further reveal that this relationship is not merely a unidirectional linear causality, but rather a dynamic, reciprocal empowerment process (Schubatzky et al., 2025). Specifically, ICT self-efficacy directly catalyzes the internalization of technological and pedagogical knowledge; meanwhile, the accumulation of TPACK fuels self-efficacy via chain mediation effects. This establishes a virtuous cycle of “knowledge growth–efficacy enhancement,” which ultimately translates into robust technological integration competence (Bai et al., 2024).

It is noteworthy that the association between ICT self-efficacy and TPACK is not fixed but varies depending on contextual and group differences. For example, a study on international Chinese pre-service teachers found that while TPACK effectively promoted the development of technology integration self-efficacy, conversely, technology integration self-efficacy could not significantly predict the development of TPACK (Wang, 2020). In contrast, subsequent research involving pre-service teachers from provincial and municipal universities with different disciplinary backgrounds found that compared to ICT perceptions, ICT self-efficacy had a more significant impact on TPACK (Wang & Zhao, 2021). This finding suggests that the psychological mechanisms formed by pre-service teachers with different backgrounds during the technology integration process may differ. Therefore, employing ICT self-efficacy as a mediating variable to deeply explore the relationships among university ICT support, pre-service teachers’ ICT self-efficacy, and TPACK is of great significance for revealing the underlying mechanisms of action. Based on the analysis of the relationships among university ICT support, ICT self-efficacy, and pre-service teachers’ TPACK, this study proposes the following research hypotheses:

**H3-1.**  *University ICT support is positively associated with pre-service teachers’ ICT self-efficacy.*

**H3-2.**  *Pre-service teachers’ ICT self-efficacy is positively associated with TPACK.*

**H3-3.**  *ICT self-efficacy mediates the relationship between university ICT support and pre-service teachers’ TPACK.*

### 2.4. The Mediating Role of AI Competency Expectancy

When external support is associated with the development of pre-service teachers’ knowledge structures and professional competencies, it often needs to be internalized through individual psychological processes. In the pathway through which AI support in education is associated with pre-service teachers’ TPACK, a key variable is AI competency expectancy. This concept originates from teachers’ need for self-fulfillment in technology learning (Liu & Zou, 2005) and reflects their overall judgment and positive vision regarding the application prospects of digital technologies (Fang et al., 2026). Research suggests that positive self-development expectancy among teachers is conducive to promoting their proactive learning and application of AI, making them more willing to actively embrace the innovations and empowerments that technologies like AI bring to education (Zhao et al., 2022). Therefore, AI competency expectancy refers to pre-service teachers’ belief in their own professional development regarding their ability to understand, master, apply, and integrate AI technologies to accomplish teaching tasks and enhance student learning. It is a concrete manifestation of teachers’ self-development expectancy in the intelligent era. It encompasses not only pre-service teachers’ confidence in operating AI tools but also a positive outlook on achieving professional development and pedagogical transformation through continuous learning and collaborative exchange in the AI era, constituting a composite belief in “being able to teach” and “being willing to learn.”

AI support in education, as a key antecedent of pre-service teachers’ AI competency expectancy, can enhance their confidence in their own AI integration capabilities by establishing a multidimensional, progressive support system that aligns technological support with their perceived competence. For example, combining teaching demonstrations with project-driven learning models can enable pre-service teachers to perceive the value and efficacy of AI technology in the process of solving specific pedagogical problems, thereby generating a motivation reinforcement effect (K. Chen et al., 2020). Inviting practicing teachers to share success stories or showcasing AI application cases in the classroom can provide pre-service teachers with referential models for emulation, allowing them to build their own sense of competence through observation (Anderson & Maninger, 2007). Meanwhile, positive feedback from mentors, peer assistance, and constructive guidance from expert-led workshops can help alleviate pre-service teachers’ anxiety when learning AI technologies, thereby consolidating their competency beliefs (Pfitzner-Eden, 2016). Finally, a supportive institutional culture can reduce negative emotions, mitigate technological anxiety, and foster a safe, positive psychological learning environment (Verano-Tacoronte et al., 2025), further enhancing AI competency expectancy. Thus, the “resources-experience-belief” chain can form a self-reinforcing mechanism for competency expectancy, transforming external support into a sustained psychological drive that serves as the cognitive and motivational foundation for the subsequent development of TPACK integration capabilities (Xu et al., 2025).

The establishment of AI competency expectancy becomes a sustained psychological driver for the development of pre-service teachers’ TPACK, and its effect is manifested by influencing learning engagement, depth of practice, and the knowledge integration process (Zeng et al., 2022). Firstly, a high level of AI competency expectancy motivates pre-service teachers to invest more energy and time in learning and practice. They demonstrate greater persistence and exploratory desire when facing technology learning tasks and are willing to try various AI tools and pedagogical strategies, rather than merely skimming the surface or choosing to avoid them. Existing research indicates that technological self-efficacy can significantly predict teachers’ intention and behavior regarding technology integration, suggesting that competency beliefs influence not only the occurrence of technology use but also its depth and breadth (Verano-Tacoronte et al., 2025; Y. Lee & Lee, 2014). Secondly, the sustained practice driven by higher competency expectancy creates conditions for the construction of TPACK. As an integrative form of knowledge, TPACK is not acquired through theoretical learning alone but needs to be continually constructed and refined through the iterative cycle of “design-practice-reflection” (G. N. H. Nguyen et al., 2022). Pre-service teachers with higher competency expectancy are more likely to actively engage in this cycle, continually linking TK with CK and exploring how to combine them with PK throughout the process. Continuous exploration and reflection help deepen the understanding of TCK and TPK, thereby promoting the integration of the three knowledge types within specific teaching contexts and ultimately enhancing TPACK.

In summary, AI support in education may be indirectly associated with TPACK through its association with pre-service teachers’ AI competency expectancy. As an external provision, the effect of AI support in education is not automatically integrated into the cognitive system of pre-service teachers; it must be processed through individual cognition and emotion to transform into lasting learning behaviors and knowledge integration. This indicates that AI support in education not only provides technical assistance but, more crucially, enhances pre-service teachers’ competency expectancy, motivating them to actively engage in AI technology-related exploration, design, practice, and reflection, thereby fostering the development of their own TPACK. Based on the above theoretical analysis and empirical findings, this study proposes the following research hypotheses:

**H4-1.**  *AI support in education is positively associated with pre-service teachers’ AI competency expectancy.*

**H4-2.**  *Pre-service teachers’ AI competency expectancy is positively associated with TPACK.*

**H4-3.**  *AI competency expectancy mediates the relationship between AI support in education and pre-service teachers’ TPACK.*

**Figure 1:**
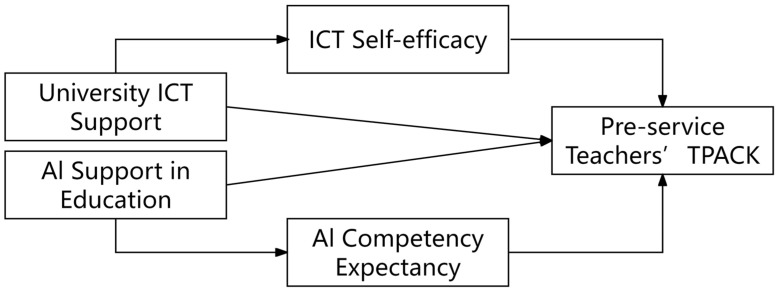
Theoretical model of the relationships among university ICT support, AI support in education, ICT self-efficacy, AI competency expectancy, and pre-service teachers’ TPACK.

Based on the above, a theoretical model depicting the mechanisms through which university ICT support and AI support in education are associated with pre-service teachers’ TPACK is constructed, as illustrated in Figure 1.

## 3. Materials and Methods

### 3.1. Participants

The data for this study were sourced from the China Teacher Cultivation and Professional Development Basic Data Platform (CTCPDBDP). This survey platform is designed to longitudinally track the pre-service teacher population in mainland China, systematically investigate their learning, training, internship, and professional development within the Chinese educational context, and focus on core issues related to the quality of the teacher education system. Within China’s pre-service teacher education system, six normal universities directly under the Ministry of Education play crucial roles in leading the development of teacher education. Meanwhile, place-based normal universities managed by provincial-level people’s governments enroll a large number of local students and primarily undertake the mission of cultivating teaching staff for local basic education. To reduce survey costs and obtain a relatively representative sample, the research team collaborated with place-based normal universities. Using a purposeful sampling method, a large-scale questionnaire survey was conducted in 2023 targeting pre-service teachers at 17 place-based normal universities located in the eastern, central, and western regions of mainland China. All participating pre-service teachers completed the questionnaire by logging into an online platform. Senior pre-service teachers are the optimal group for investigating TPACK levels, as they have generally completed their foundational professional coursework and initial teaching practice, resulting in a TPACK structure that has begun to stabilize. Based on the availability of data for the study’s core variables within the CTCPDBDP, this study limited the sample to third-year (junior-year) pre-service teachers, initially obtaining 12,181 questionnaires. After manual screening to invalidate duplicate submissions, patterned responses, questionnaires with high missing rates, and those containing logical errors, 11,818 valid questionnaires were ultimately confirmed. The basic sample information is presented in Table 1.

### 3.2. Variables and Measures

The measurement instruments used in this study were adapted from relevant authoritative scales. Their development considered the dual identity of pre-service teachers as both “students” and “future teachers.” Multiple experts were invited to revise the items and evaluate their content validity before finalizing the scales. In this study, all items across the five scales employed a five-point Likert scale for measurement. Meanwhile, the sources of the measurement scales, the number of items, and the reliability for the five variables are shown in Table 2. Additionally, the study collected demographic information on the participants. Considering that gender differences may correlate with ICT self-efficacy and AI competency expectancy, household registration type (hukou) may reflect the impact of urban-rural resource disparities on perceptions of university ICT support and AI support in education, and pre-service teacher identity type may influence TPACK development trajectories, we selected gender, household registration type, and identity type as control variables.

#### 3.2.1. Pre-Service Teachers’ TPACK

The pre-service teachers’ TPACK scale was primarily adapted from the scale developed by Wang and Zhao (2021). It consists of 5 items. Example items include: “I can use information technology to conduct formative and summative assessments of subject teaching”; “I can, according to syllabus requirements, use appropriate technologies (e.g., mind mapping software) to help students construct subject knowledge”; and “I can appropriately integrate subject knowledge, information technology, and pedagogical knowledge to design student-centered learning activities.” In this study, TPACK is therefore operationalized as self-reported TPACK competence. The scale captures pre-service teachers’ perceived capacity to integrate technology, pedagogy, and content knowledge; it should not be read as a direct performance-based assessment of classroom technology integration. Regarding reliability and validity, the pre-service teachers’ TPACK scale demonstrated a Cronbach’s α coefficient of 0.909. Confirmatory Factor Analysis (CFA) results indicated that, as the chi-square statistic (χ^2^) is highly sensitive to sample size, the high χ^2^/df ratio of 63.892 is a common occurrence with the very large sample size of this study (N = 11,818) (Bagozzi, 1981; Bagozzi & Yi, 1988). Other model fit indices, which are relatively less sensitive to sample size, all met the respective criteria: RMSEA = 0.073; RMR = 0.008; GFI = 0.989; TLI = 0.983; CFI = 0.992. The standardized factor loadings for the 5 items ranged from 0.791 to 0.841. The average variance extracted (AVE) was 0.668, and the composite reliability (CR) was 0.910. Overall, the pre-service teachers’ TPACK scale exhibits high reliability and validity.

#### 3.2.2. University ICT Support

The university ICT support scale was primarily adapted from the scale developed by Wang and Zhao (2021). It consists of 3 items: “The university offers a reasonable curriculum in information technology, which meets my learning needs in this area”; “The university provides sufficient electronic resources (e.g., software, databases), enabling me to complete related learning tasks smoothly”; and “I can conveniently obtain technical assistance from university teachers or technical staff.” In terms of reliability, the university ICT support scale demonstrated a Cronbach’s α coefficient of 0.865. Regarding validity, as university ICT support is a unidimensional construct measured by a three-item scale with limited model degrees of freedom (df = 1) and a large sample size (N = 11,818), the reference value of traditional fit indices (e.g., χ^2^/df, RMSEA) is constrained. Therefore, the analysis focused on convergent validity. CFA results showed that the standardized factor loadings for the 3 items ranged from 0.858 to 0.915. The AVE was 0.776, and the CR was 0.912. Overall, the university ICT support scale exhibits high reliability and convergent validity.

#### 3.2.3. ICT Self-Efficacy

The ICT self-efficacy scale was primarily adapted from the scale developed by Wang and Zhao (2021). It consists of 4 items. Example items include: “I can easily learn new information technologies”; “When I try my best, I can solve technology-related problems on my own”; and “I am capable of using information technology to achieve teaching objectives.” In terms of reliability and validity, the ICT self-efficacy scale demonstrated a Cronbach’s α coefficient of 0.873. CFA results indicated that, as the χ^2^ is highly sensitive to sample size, the high χ^2^/df ratio of 61.838 is a common occurrence with the very large sample size of this study (N = 11,818) (Bagozzi, 1981; Bagozzi & Yi, 1988). Other model fit indices, which are relatively less sensitive to sample size, all met the respective criteria: RMSEA = 0.072; RMR = 0.006; GFI = 0.997; TLI = 0.984; CFI = 0.997. The standardized factor loadings for the 4 items ranged from 0.788 to 0.836. The AVE was 0.649, and the CR was 0.881. Overall, the ICT self-efficacy scale exhibits high reliability and validity.

#### 3.2.4. AI Support in Education

The AI support in education scale was primarily adapted from the scale developed by Nazaretsky et al. (2022). It consists of 7 items. Example items include: “Helping teachers conduct formative assessment of complex tasks (e.g., open-ended questions) and provide real-time personalized feedback”; “Creating intelligent apps to serve as learning companions or teaching assistants for students”; and “Simulating teaching practice scenarios to help pre-service teachers and in-service teachers improve their teaching performance.” In this study, AI support in education is operationalized as pre-service teachers’ perceived availability and educational value of AI-enabled support scenarios in teacher education. The items refer to concrete AI-supported functions such as formative feedback, personalized learning paths, learning companions, learning-difficulty diagnosis, classroom management, lesson preparation, and simulated teaching practice; the scale is not an objective institutional audit of AI infrastructure or a log of actual AI use. In terms of reliability and validity, the scale demonstrated a Cronbach’s α coefficient of 0.918. CFA results indicated that, given the χ^2^ high sensitivity to sample size, the high χ^2^/df ratio of 64.372 is expected with this study’s very large sample (N = 11,818) (Bagozzi, 1981; Bagozzi & Yi, 1988). Other model fit indices, which are relatively less sensitive to sample size, all met the respective criteria: RMSEA = 0.073; RMR = 0.012; GFI = 0.978; TLI = 0.974; CFI = 0.983. The standardized factor loadings for the 7 items ranged from 0.693 to 0.836. The AVE was 0.620, and the CR was 0.919. Overall, the AI support in education scale exhibits high reliability and validity.

#### 3.2.5. AI Competency Expectancy

The AI competency expectancy scale was primarily developed with reference to the scale items by Zhao et al. (2022). It consists of 5 items. Example items include: “I am willing to strive for further training opportunities in the educational application of technologies such as artificial intelligence and big data”; “I hope to acquire knowledge and skills in areas like educational artificial intelligence and educational big data”; and “I expect to communicate or exchange ideas with experts and other teachers in fields such as educational artificial intelligence.” In terms of reliability and validity, the scale demonstrated a Cronbach’s α coefficient of 0.913. CFA results indicated that, given the χ^2^ high sensitivity to sample size, the high χ^2^/df ratio of 63.130 is expected with this study’s very large sample (N = 11,818) (Bagozzi, 1981; Bagozzi & Yi, 1988). Other model fit indices, which are relatively less sensitive to sample size, all met the respective criteria: RMSEA = 0.073; RMR = 0.007; GFI = 0.992; TLI = 0.984; CFI = 0.994. The standardized factor loadings for the 5 items ranged from 0.769 to 0.858. The AVE was 0.670, and the CR was 0.910. Overall, the AI competency expectancy scale exhibits high reliability and validity.

### 3.3. Data Analysis

This study employed IBM SPSS Amos (v26.0; IBM Corp., Armonk, NY, USA), IBM SPSS Statistics (v27.0; IBM Corp., Armonk, NY, USA), and R (v4.4.2; R Core Team, Vienna, Austria) as the primary data-analytic tools, following a systematic procedure. First, because pre-service teachers were nested within 17 universities, null random-intercept models were estimated to calculate intraclass correlation coefficients (ICCs) for the core constructs, and the main predictive model was re-estimated with university-clustered robust standard errors as a robustness check. Second, SPSS 27.0 and AMOS 26.0 were used to assess reliability, convergent validity, and discriminant validity. Third, AMOS 26.0 was used to test common method bias, and SPSS 27.0 was used for descriptive and correlation analyses. Fourth, multi-group CFA was conducted to test configural, metric, and scalar measurement invariance across gender, household registration, tuition-free status, and major type. Fifth, hierarchical regression and quantile regression were conducted with gender, household registration, and identity type as control variables; Wald tests were used to formally compare selected quantile coefficients. Finally, a structural equation model was constructed using AMOS 26.0, and the Bootstrap method was applied to examine model-conform indirect associations through ICT self-efficacy and AI competency expectancy.

## 4. Results

### 4.1. Multilevel Structure and Common Method Bias Test

Before testing the main models, we examined the university-level nesting structure. The intraclass correlation coefficients (ICCs; Table A1) were low for all core constructs: university ICT support (ICC = 0.016), AI support in education (ICC = 0.010), ICT self-efficacy (ICC = 0.013), AI competency expectancy (ICC = 0.011), and self-reported TPACK (ICC = 0.015). These values indicate limited between-university variance. To address the remaining dependency risk, the main predictive model was also re-estimated using university-clustered robust standard errors; the core coefficients remained statistically significant, including university ICT support (β = 0.137, clustered SE = 0.007, *p* < 0.001), AI support in education (β = 0.218, clustered SE = 0.012, *p* < 0.001), ICT self-efficacy (β = 0.410, clustered SE = 0.011, *p* < 0.001), and AI competency expectancy (β = 0.126, clustered SE = 0.009, *p* < 0.001). These checks supported the use of the single-level SEM while acknowledging the nested sampling design.

This study then employed two methods to examine CMB. First, a single-factor CFA was conducted. The fit indices for the single-factor model [χ^2^/df = 252.522, RMR = 0.068, RMSEA = 0.146, GFI = 0.515, CFI = 0.683] all failed to meet acceptable standards. In contrast, the fit indices for the five-factor model [χ^2^/df = 18.033; RMR = 0.014; RMSEA = 0.038; GFI = 0.967; CFI = 0.979] all met the criteria for good model fit (L. Hu & Bentler, 1999; Wu, 2018). Furthermore, as shown in Table 3, the square roots of the AVE for all five variables were greater than the Pearson correlation coefficients between any pair of variables, providing additional evidence for the good discriminant validity of the scales used in this study (Hair et al., 2011). Subsequently, the unmeasured latent method factor approach was applied to further test for CMB. A common method latent factor was added to the original five-factor model. The results indicated that the six-factor model did not yield marked improvement in fit indices compared to the five-factor model [ΔRMSEA = 0.002, ΔRMR = 0.001, ΔNFI = 0.002, ΔTLI = 0.002, ΔCFI = 0.003]. Based on the combined results of these two tests, it is concluded that the data in this study do not exhibit a serious CMB problem.

Measurement invariance was also examined through multi-group CFA (Table A2). Across gender, household registration, identity type, and major type, the configural, metric, and scalar models all showed acceptable fit (CFI = 0.976~0.979, RMSEA = 0.037~0.041, SRMR = 0.022~0.025). Changes in fit were very small from configural to metric and from metric to scalar models (ΔCFI ≤ 0.001; ΔRMSEA ≤ 0.001), supporting configural, metric, and scalar invariance across these key subgroups. These results indicate that the scales operated comparably across major demographic and subject-area groups before the distributional analyses were interpreted.

### 4.2. Direct Association Test Results

This study employed hierarchical regression analysis to examine the direct association. We entered pre-service teachers’ gender, household registration, and identity type as control variables. The results are presented in Table 4. Firstly, university ICT support was positively associated with ICT self-efficacy (Model 9: β = 0.630, *p* < 0.001) and with pre-service teachers’ TPACK (Model 2: β = 0.528, *p* < 0.001). ICT self-efficacy was also positively associated with pre-service teachers’ TPACK (Model 3: β = 0.650, *p* < 0.001). Secondly, AI support in education was positively associated with AI competency expectancy (Model 11: β = 0.765, *p* < 0.001) and with pre-service teachers’ TPACK (Model 5: β = 0.604, *p* < 0.001). AI competency expectancy was also positively associated with pre-service teachers’ TPACK (Model 6: β = 0.546, *p* < 0.001). Finally, upon introducing ICT self-efficacy into the regression equation for the association of university ICT support on TPACK, the direct association of university ICT support decreased (Model 4), providing initial evidence that ICT self-efficacy partially mediates the association between university ICT support and pre-service teachers’ TPACK. Similarly, introducing AI competency expectancy into the regression equation for the association of AI support in education with TPACK also decreased the direct association of AI support in education (Model 7), providing initial evidence that AI competency expectancy also partially mediates the association between AI support in education and pre-service teachers’ TPACK. These results support Hypotheses 1, 2, 3-1, 3-2, 4-1, and 4-2, and provide initial support for Hypotheses 3-3 and 4-3.

### 4.3. Quantile-Specific Association Patterns

**Figure 2:**
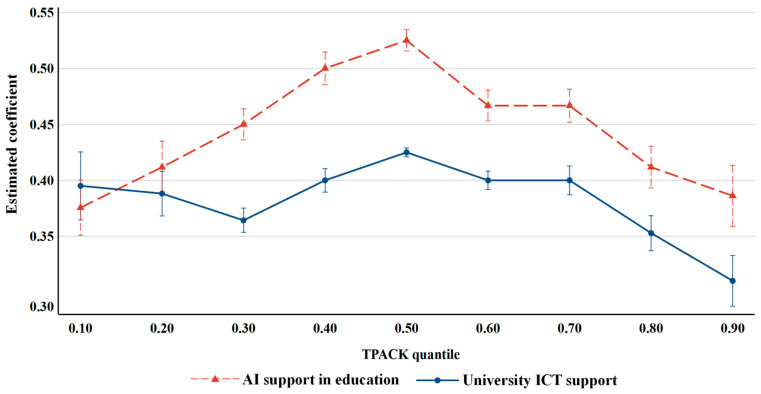
Quantile-specific coefficients with 95% confidence intervals.

To explore whether the associations of university ICT support and AI support in education with pre-service teachers’ TPACK were heterogeneous across different quantile levels, this study employed quantile regression models with gender, household registration type, and tuition-free status included as control variables. The quantile points ranged from 0.10 to 0.90, and the results are presented in Table 5. The corresponding coefficient patterns are visualized in Figure 2. Both university ICT support and AI support in education were positively associated with pre-service teachers’ TPACK at all quantile levels, but the magnitude of these associations varied across quantiles. The university ICT support coefficients showed a modestly fluctuating descriptive pattern and were strongest at the 0.50 quantile (β = 0.425, *p* < 0.001), before attenuating at upper quantiles. The AI support coefficients increased from the lower quantiles to the 0.50 quantile (β = 0.525, *p* < 0.001) and then attenuated at higher quantiles. We therefore interpret these patterns as distributional heterogeneity rather than as strict developmental trajectories or a statistical ceiling effect. Formal Wald tests (Table A3) further supported selected coefficient differences: for university ICT support, Q0.10 vs. Q0.50 (F = 3.947, *p* = 0.047), Q0.50 vs. Q0.90 (F = 109.394, *p* < 0.001), and Q0.10 vs. Q0.90 (F = 21.508, *p* < 0.001) differed significantly; for AI support in education, Q0.10 vs. Q0.50 (F = 150.795, *p* < 0.001) and Q0.50 vs. Q0.90 (F = 111.079, *p* < 0.001) differed significantly, whereas Q0.10 vs. Q0.90 did not (F = 0.357, *p* = 0.550). These coefficient differences suggest that generalized ICT and AI support may be most educationally meaningful when aligned with different quantile levels of self-reported TPACK: foundational scaffolding at lower quantile levels, guided AI-supported design practice around the median quantile, and more specialized disciplinary or ethical design challenges at higher quantile levels.

### 4.4. Mediating Association Test Results

**Figure 3:**
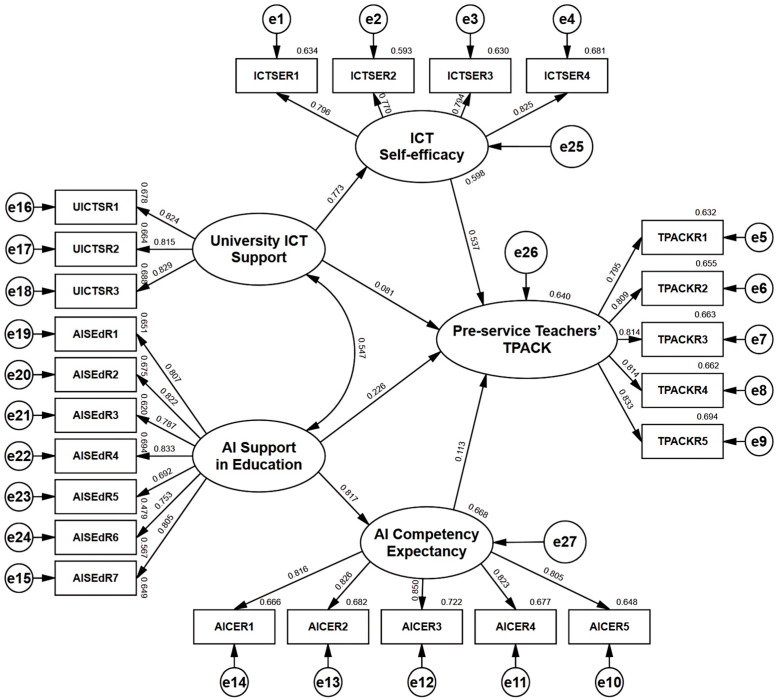
Structural model of the relationships among university ICT support, AI support in education, ICT self-efficacy, AI competency expectancy, and pre-service teachers’ TPACK.

To further examine the synergistic effect of university ICT support and AI support in education on pre-service teachers’ TPACK and to assess the differences in their mediating roles, this study constructed a structural model containing two independent variables and two mediating variables based on structural equation modeling, as shown in Figure 3. This approach was taken to enhance the accuracy and robustness of the findings. Following a series of steps including model specification, identification, and parameter estimation, the fit of the structural model was evaluated. The results show that, given the χ^2^ high sensitivity to sample size, the high χ^2^/df ratio of 20.195 is expected with this study’s very large sample (N = 11,818) (Bagozzi, 1981; Bagozzi & Yi, 1988). Other model fit indices, which are relatively less sensitive to sample size, all met the accepted standards: RMSEA = 0.040, RMR = 0.026, GFI = 0.964, TLI = 0.974, CFI = 0.977. This indicates that the dual-independent-variable dual-mediator model demonstrates a good fit with the empirical data.

Simultaneously, this study employed the Bootstrap method to test the indirect associations. The confidence intervals for the indirect associations of ICT self-efficacy and AI competency expectancy were estimated through 5000 bootstrap resamples. The results are shown in Table 6. Specifically, the bias-corrected and 95th percentile confidence intervals for both ICT self-efficacy and AI competency expectancy did not contain zero, indicating that both indirect associations were significant and both played a partial mediating role. This verifies Hypotheses 2-3 and 3-3. Among them, the mediating effect size for ICT self-efficacy was 0.415, accounting for 83.67%; the mediating effect size for AI competency expectancy was 0.093, accounting for 29.25%. Regarding the results of the contrast effect tests, the total contrast effect, direct contrast effect, and mediating contrast effect were 0.178, −0.144, and 0.322, respectively. Their confidence intervals did not contain zero. This indicates that, compared to AI support in education, university ICT support has a significantly higher total association, a significantly lower direct association, and a significantly higher mediating association on pre-service teachers’ TPACK.

## 5. Discussion

Two forms of institutional support shaped pre-service teachers’ TPACK, but not in the same way. University ICT support showed a modestly fluctuating quantile pattern, AI support in education showed stronger coefficients around the median than at lower or upper quantiles, ICT self-efficacy carried most of the indirect ICT pathway, and AI competency expectancy played a smaller bridging role in the AI pathway.

**Figure 4:**
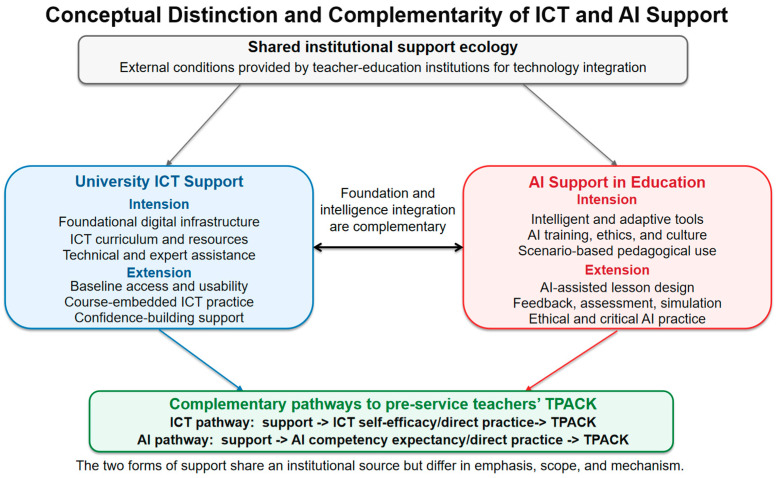
Conceptual distinction and complementarity of university ICT support and AI support in education.

To clarify the conceptual distinction behind these empirical patterns, Figure 4 summarizes the shared institutional source, different emphases, and complementary mechanisms of university ICT support and AI support in education. ICT support is positioned as the foundational layer of digital access, curriculum resources, and technical assistance, whereas AI support extends this foundation into intelligent, adaptive, and ethically guided pedagogical practice. The two forms of support are therefore not competing categories but complementary layers of institutional support for TPACK development.

### 5.1. Theoretical Contributions

Institutional support is associated with TPACK through different mediated pathways. University ICT support works mainly through psychological empowerment. Course resources, technical tutoring, and digital infrastructure build technological confidence first and support deeper knowledge integration afterward. Self-efficacy shapes behavioral choices and effort investment (Bandura et al., 1999). Teachers with stronger ICT self-efficacy also report more positive attitudes toward technology integration (Holden & Rada, 2011), and multiple pre-service studies link self-efficacy to TPACK (Abbitt, 2011; Bilici et al., 2013; M.-H. Lee & Tsai, 2010). The stronger indirect path fits a belief-before-practice process: external resources matter most after they have been internalized as efficacy beliefs. AI support in education follows a different route. Practice-based teacher-education strategies raise preservice teachers’ TPACK (Baran et al., 2019), and AI tools create immediate opportunities to rehearse feedback, lesson adaptation, and instructional design (Holmes et al., 2022; Heine & König, 2025). AI competency expectancy still plays a partial bridging role. That pattern fits the Technology Acceptance Model (Davis, 1989) and prior evidence that pre-service teachers’ technology use is tightly connected to their TPACK development (Pamuk, 2012). Different support types thus reach the same developmental outcome through different channels, extending both the TPACK framework (Koehler & Mishra, 2006) and the AI-TPACK model (Celik, 2023).

Support effectiveness also shifted across the TPACK distribution. University ICT support was strongest at lower-to-median quantile levels and weaker at higher quantile levels. Foundational resources appear to matter most while pre-service teachers are still building a usable integration repertoire. Prior TPACK research similarly links weaker constructivist and technology-integration orientations with lower levels of technology-integration readiness (Koh et al., 2014). Authentic ICT-rich learning experiences and teacher-education support can strengthen initial adoption and use intentions (Tondeur et al., 2019; Valtonen et al., 2015). At higher quantile levels, generalized provision gives way to subject-specific design opportunities. That reading fits evidence that lesson-plan quality and TPACK are more tightly linked when technology use is embedded in instructional contexts (Schmid et al., 2021). The AI-support pattern, with stronger coefficients around the median, implies a comparable readiness-support match. Around the median quantile level, pre-service teachers may be ready to experiment with AI without yet having moved beyond generalized support, whereas at lower quantile levels they may still face knowledge-integration constraints (Koehler & Mishra, 2009). At higher quantile levels, generic AI support adds less value.

Figure 2 clarifies this interpretation visually. The ICT-support coefficients fluctuate within a narrower band and then decline after the upper-middle quantiles, indicating that general ICT provision becomes less differentiated at higher quantile levels of self-reported TPACK. By contrast, the AI-support coefficients rise from the lower quantiles to the median and then decline, showing that AI support is most powerful around the median quantile, where pre-service teachers may already possess enough technological-pedagogical grounding to engage in AI-mediated design but have not yet reached a point at which generic AI resources add limited additional pedagogical leverage.

At higher quantile levels, pre-service teachers appear to need more specialized design tasks than the current support environment provides. Recent studies in teacher education likewise report uneven AI literacy, uneven confidence in AI-driven applications, and a need for guided reflection when preservice teachers use generative AI in coursework (Heine & König, 2025; Laru et al., 2025). Quantile-sensitive task differentiation therefore fits the evidence better than a uniform support model.

Self-efficacy and technology acceptance also intersect rather than operating as separate explanatory tracks. TPACK encompasses both integration and transformation perspectives (Angeli & Valanides, 2009). Conventional ICT support is closer to integration because it strengthens readiness to connect technology with existing pedagogical and content knowledge. AI support is closer to transformation because it pushes preservice teachers to rework feedback, adaptation, and design decisions around AI-enabled tools (Celik, 2023; L. Chen et al., 2020). Because TPACK is personalized, contextualized, and constructive (Koehler & Mishra, 2006), the balance between cognitive and experiential pathways is likely to shift with the form of technology support available to learners.

### 5.2. Practical Implications

General-purpose ICT provision loses leverage once TPACK reaches the upper part of the distribution. Teacher education programs should therefore complement foundational ICT support with subject-specific technology integration opportunities, such as discipline-based instructional design projects and micro-teaching with embedded technology feedback (Baran et al., 2019). Because ICT self-efficacy mediates more strongly than the direct resource pathway, efficacy-building should be embedded in the ordinary design-practice-reflection cycle of initial teacher education rather than treated as a separate motivational activity. In this context, mastery experiences can be created when candidates design a technology-supported lesson, teach a micro-lesson, revise the lesson after feedback, and see how the revision improves the instructional design. Peer modeling occurs when candidates observe classmates or mentor teachers demonstrate how a digital tool supports a specific pedagogical move and then adapt that model in their own subject area. Formative confidence assessments can be organized as brief self-rating, reflection, and feedback cycles before and after technology-rich tasks, helping candidates identify which integration decisions they can handle independently and which still require support.

AI support presents a different practical pattern. Readiness should be assessed before AI-based pedagogical tools are deployed at scale. At lower quantile levels of self-reported TPACK, pre-service teachers may benefit from scaffolded introductions to AI rather than direct immersion, whereas at higher quantile levels they may need more complex AI application challenges, such as designing AI-augmented curricula, to sustain further growth. The stronger direct effect in the AI pathway favors hands-on tool use over lecture-only AI literacy courses. Earlier evidence on ICT integration remains valuable because authentic technology-rich learning experiences can strengthen preservice teachers’ intention to use digital tools for teaching (Valtonen et al., 2015). In the AI-enabled context, however, authenticity needs to be extended from tool use to generative, adaptive, and ethically accountable design. For example, pre-service teachers can use generative AI to co-design curriculum-aligned digital stories, create and critique multimodal materials, document prompt revisions, and check copyright, attribution, and developmental appropriateness (Onbasili, 2026). They can also engage in GenAI teaching simulations in which AI-powered student avatars allow candidates to practice eliciting student thinking and receive formative evidence about instructional strengths and areas for growth (Mikeska et al., 2025). Course-level collaborative projects, such as co-authoring open educational resources on responsible GenAI use, provide another route for combining AI literacy, instructional design, peer feedback, and ethical reflection (MacDowell et al., 2024). These examples extend rather than replace the earlier ICT-rich learning logic: candidates still learn through tool-mediated pedagogical practice, but the tasks now require curriculum alignment, prompt iteration, formative evidence, and ethical judgment.

Furthermore, this study employed same-source, single-time-point self-report measures to survey TPACK. This approach essentially captures pre-service teachers’ subjective competence beliefs rather than the integrative professional competence demonstrated in actual teaching practice, which may blur the boundary between beliefs regarding technology integration and actual technology integration capability. Consequently, the positive associations identified among ICT support, AI support, and pre-service teachers primarily reflect their psychological preparedness regarding TPACK, rather than being equated with their actual TPACK proficiency. Additionally, while the findings suggest that university ICT support is positively associated with TPACK primarily through psychological empowerment and that AI support in education is positively associated with TPACK primarily through competency expectancy, these association pathways are likely dynamic. Thus, the results of this study fundamentally represent an explanation of the psychological association mechanisms among these variables.

### 5.3. Limitations and Future Directions

Causal ordering remains uncertain because the design is cross-sectional. Longitudinal or experimental work is needed to clarify temporal dynamics. All variables were measured through self-report instruments, so some exposure to common method bias persists despite the controls applied, including Harman’s single-factor test and unmeasured latent method factor analysis. The sample was drawn exclusively from third-year pre-service teachers in Chinese normal universities, which limits generalizability to other educational contexts and career stages. Extending the evidence base to in-service teachers and cross-cultural settings, and using mixed methods, can clarify how institutional technology support is translated into TPACK growth. Furthermore, future research should integrate longitudinal tracking data with objective teaching behavior observation data to disentangle the confounding between pre-service teachers’ subjective competence beliefs and their actual capabilities regarding TPACK.

Given these limitations, the practical implications of this study should be understood as design-oriented directions that require further empirical examination. In particular, the associations observed among perceived AI support, AI competency expectancy, and self-reported TPACK suggest that future teacher-education research should examine how AI-supported learning environments can be organized to strengthen pre-service teachers’ professional judgment rather than bypass it. A human-in-the-loop interpretation further specifies how these implications should be translated into teacher-education design, positioning AI as a partner for feedback and simulation rather than an autonomous pedagogical decision-maker. This framing emphasizes that professional judgment, ethical responsibility, and subject-specific pedagogy remain firmly in the hands of human teachers while leveraging AI to expand opportunities for practice and formative guidance.

## 6. Conclusions

Survey data from 11,818 pre-service teachers across 17 local normal universities in China indicate that perceived university ICT support and perceived AI support in education are associated with self-reported TPACK through different structural routes. University ICT support was positively associated with self-reported TPACK, and its quantile coefficients showed a modestly fluctuating descriptive pattern: strongest around the median, attenuating at higher quantiles, and fluctuating mildly at lower quantiles. AI support in education was also positively associated with self-reported TPACK, with coefficients that were largest around the median quantile and attenuated at lower and higher quantiles.

ICT self-efficacy and AI competency expectancy did not contribute in the same way in the hypothesized model. ICT self-efficacy formed a significant indirect pathway in the university ICT support-TPACK association, and the indirect association substantially exceeded the direct association. AI competency expectancy also formed a significant indirect pathway in the AI support-TPACK association, but the direct association remained dominant. Given the cross-sectional self-report design, these pathways should be interpreted as model-conform indirect associations rather than evidence of temporal or causal mediation.

Neither support pathway was uniform across mechanisms or across the conditional TPACK distribution. ICT support worked mainly through psychological empowerment, whereas AI support in education worked more directly through experiential engagement; both pathways also varied across quantile levels of self-reported TPACK. Teacher education programs therefore need differentiated support that combines efficacy-building for ICT integration with hands-on practice for AI-enabled pedagogical innovation. At lower quantile levels, this may mean structured exposure to AI concepts, ethical risks, and simple prompt-based teaching cases; around the median quantile, guided design tasks such as lesson-plan revision, AI-assisted formative assessment, and micro-teaching simulations may be more appropriate; at higher quantile levels, open-ended AI-enabled pedagogical innovation, including discipline-specific curriculum design, student-data-informed feedback routines, and critical evaluation of AI outputs, may be more suitable. Differentiation should therefore refer not only to the amount of support provided, but also to the complexity, autonomy, ethical responsibility, and subject specificity of the AI-enabled teaching task.

## Figures and Tables

**Table 1:** Demographic Characteristics of Study Participants.

Variables	Categories	Number	Percentage/%
Gender	Male	2612	22.10
	Female	9206	77.90
Household Registration	Rural	8137	68.85
	Urban	3681	31.15
Ethnic Group	Han ethnicity	9741	82.43
	Ethnic minorities	2077	17.57
Directional Employment (Yes/No)	Yes	1467	12.41
	No	10,351	87.59
Identity Type	Tuition—free	2120	17.94
	Non—tuition—free	9698	82.06
Highest Education Level of Parents	Postgraduate	118	1.00
	Junior College/Bachelor’s degree	2278	19.28
	Senior High School/Junior College	3117	26.38
	Junior High School and below	6305	53.35
Major Type	Humanities and Social Sciences	3032	25.65
	Science and Engineering	4162	35.22
	Arts and Sports	2387	20.20
	Comprehensive	2237	18.93
Region	Eastern region	1746	14.77
	Central region	4325	36.60
	Western region	5747	48.63
Family Residence Location	Urban main district	3057	25.87
	Urban—rural fringe area	1643	13.90
	Town center area	1056	8.94
	Town—rural fringe area	1100	9.31
	Rural area	4962	41.99
Parents as Teachers (Yes/No)	Yes	953	8.06
	No	10,865	91.94

**Table 2:** Source, Number of Items, and Reliability of Measurement Scales.

Variables	Source	Items (n)	Standardized Factor Loadings	AVE	CR	Cronbach’s α
University ICT Support	Wang and Zhao (2021)	3	0.858~0.915	0.776	0.912	0.865
Al Support in Education	Nazaretsky et al. (2022)	7	0.693~0.836	0.620	0.919	0.918
ICT Self-efficacy	Wang and Zhao (2021)	4	0.788~0.836	0.649	0.881	0.873
Al Competency Expectancy	Zhao et al. (2022)	5	0.769~0.858	0.670	0.910	0.913
TPACK	Wang and Zhao (2021)	5	0.791~0.841	0.668	0.910	0.909

**Table 3:** Results of Descriptive Statistics, Correlations, and Discriminant Validity Analyses.

Variables	M	S.D.	1	2	3	4	5
University ICT Support (1)	3.921	0.729	0.826				
AI Support in Education (2)	3.908	0.625	0.459 **	0.787			
ICT Self-efficacy (3)	3.784	0.696	0.659 **	0.471 **	0.796		
AI Competency Expectancy (4)	3.913	0.643	0.420 **	0.743 **	0.443 **	0.824	
TPACK (5)	3.836	0.665	0.578 **	0.567 **	0.678 **	0.528 **	0.817

Note: ** *p* < 0.01; The diagonal represents the square root of the average variance extracted (AVE) of the five variables; The lower triangular area represents the Pearson correlation coefficients among the five variables.

**Table 4:** Results of Hierarchical Regression Analysis of University ICT Support and AI Support in Education on Pre-service Teachers’ TPACK.

Variables		Pre-Service Teachers’ TPACK							ICT Self-Efficacy		Al Competency Expectancy	
		Model 1	Model 2	Model 3	Model 4	Model 5	Model 6	Model 7	Model 8	Model 9	Model 10	Model 11
Intercept		3.868 ***	1.807 ***	1.404 ***	1.136 ***	1.489 ***	1.717 ***	1.261 ***	3.793 ***	1.332 ***	3.938 ***	0.927 ***
Control Variables	Gender	−0.008	0.001	−0.062 ***	−0.046 ***	0.023	0.021	0.026 *	0.082 ***	0.094 ***	−0.054 ***	−0.015
	Household Registration	−0.047 ***	−0.056 ***	−0.019	−0.029 **	−0.031 **	−0.036 **	−0.031 **	−0.044 **	−0.054 ***	−0.020	0.001
	Identity Type	0.011	−0.011	0.003	−0.004	0.008	0.008	0.007	0.013	−0.014	0.006	0.002
Independent Variables	University ICT Support		0.528 ***		0.210 ***					0.630 ***		
	Al Support in Education					0.604 ***		0.416 ***				0.765 ***
Mediating Variables	ICT Self-efficacy			0.650 ***	0.504 ***							
	Al Competency Expectancy						0.546 ***	0.246 ***				
*R* ^2^		0.001	0.335	0.462	0.491	0.322	0.279	0.348	0.003	0.439	0.001	0.553
Adjusted *R*^2^		0.001	0.335	0.461	0.491	0.322	0.279	0.347	0.003	0.439	0.001	0.552
*F*		4.534	1490.357	2531.220	2282.397	1405.770	1145.264	1259.551	13.024	2308.213	5.742	3647.446

Note: * *p* < 0.05, ** *p* < 0.01, *** *p* < 0.001.

**Table 5:** Quantile Regression Analysis Results.

Variables	Quantile								
	0.10	0.20	0.30	0.40	0.50	0.60	0.70	0.80	0.90
Intercept	0.195 * (0.076)	0.388 *** (0.049)	0.486 *** (0.032)	0.300 *** (0.033)	0.200 *** (0.019)	0.533 *** (0.036)	0.667 *** (0.031)	1.177 *** (0.048)	1.635 *** (0.059)
Control Variables	YES	YES	YES	YES	YES	YES	YES	YES	YES
University ICT Support	0.395 *** (0.016)	0.388 *** (0.049)	0.364 *** (0.006)	0.400 *** (0.005)	0.425 *** (0.002)	0.400 *** (0.004)	0.400 *** (0.007)	0.353 *** (0.008)	0.310 *** (0.012)
Al Support in Education	0.376 *** (0.013)	0.412 *** (0.012)	0.450 *** (0.007)	0.500 *** (0.007)	0.525 *** (0.005)	0.467 *** (0.007)	0.467 *** (0.008)	0.412 *** (0.010)	0.386 *** (0.014)

Note: * *p* < 0.05, *** *p* < 0.001; Standard errors (SE) are in parentheses; Control variables include gender, household registration, and identity type.

**Table 6:** Direct, Mediating, and Total Effects of the Model.

Model Path	Estimate	Product of Coefficients		Bias-Corrected 95%CI		Percentile 95%CI		*p*
		SE	Z	Lower	Upper	Lower	Upper	
Total: U-ICT-S →PT-TPACK	0.496	0.012	41.333	0.472	0.520	0.472	0.520	0.000
Total: AIS-Ed →PT-TPACK	0.318	0.012	26.500	0.293	0.342	0.293	0.342	0.000
Direct: U-ICT-S →PT-TPACK	0.081	0.018	4.500	0.046	0.115	0.046	0.116	0.000
Direct: AIS-Ed →PT-TPACK	0.226	0.020	11.300	0.187	0.264	0.186	0.263	0.000
Indirect: U-ICT-S →ICT-SE→PT-TPACK	0.415	0.014	29.643	0.387	0.443	0.387	0.443	0.000
Indirect: AIS-Ed →AI-CE→PT-TPACK	0.093	0.015	6.200	0.063	0.122	0.064	0.123	0.000
Contrast Total Effect	0.178	0.023	7.739	0.133	0.223	0.133	0.224	0.000
Contrast Direct Effect	−0.144	0.029	−4.966	−0.202	−0.088	−0.200	−0.087	0.000
Contrast Indirect Effect	0.322	0.022	14.636	0.279	0.364	0.279	0.364	0.000

Note: U-ICT-S = University ICT Support; AIS-Ed = AI Support in Education; ICT-SE = ICT Self-efficacy; AI-CE = AI Competency Expectancy; PT-TPACK = Pre-service Teachers’ TPACK; The arrow (→) indicates a directed model path.

## Data Availability

The data that support the findings of this study are available on request from the corresponding author.

## Appendix A. Measurement Validation and Statistical Diagnostics

**Table A1:** Intraclass correlation coefficients for the core constructs.

Construct	University Variance	Residual Variance	ICC
University ICT support	0.009	0.524	0.016
AI support in education	0.004	0.386	0.010
ICT self-efficacy	0.006	0.479	0.013
AI competency expectancy	0.004	0.409	0.011
TPACK	0.007	0.436	0.015

Note: ICC values were estimated from null random-intercept models with university as the grouping variable.

**Table A2:** Measurement invariance tests across key subgroups.

Grouping	Model	CFI	TLI	RMSEA	SRMR	Delta CFI	Delta RMSEA
Gender	Configural	0.978	0.975	0.039	0.022		
Gender	Metric	0.978	0.976	0.039	0.023	0.000	−0.001
Gender	Scalar	0.977	0.976	0.038	0.023	−0.001	0.000
Household registration	Configural	0.978	0.975	0.039	0.022		
Household registration	Metric	0.978	0.976	0.038	0.022	0.000	−0.001
Household registration	Scalar	0.978	0.977	0.038	0.023	0.000	−0.001
Identity type	Configural	0.979	0.976	0.039	0.022		
Identity type	Metric	0.979	0.976	0.038	0.022	0.000	−0.001
Identity type	Scalar	0.979	0.977	0.037	0.022	0.000	−0.001
Major type	Configural	0.977	0.973	0.041	0.023		
Major type	Metric	0.977	0.975	0.039	0.024	0.000	−0.001
Major type	Scalar	0.976	0.975	0.039	0.025	−0.001	0.000

Note: Delta values compare each model with the immediately preceding invariance model.

**Table A3:** Wald tests of differences between selected quantile coefficients.

Comparison	Predictor	Wald F	*p*
Q0.1 vs. Q0.5	University ICT support	3.947	0.047
Q0.1 vs. Q0.5	AI support in education	150.795	<0.001
Q0.5 vs. Q0.9	University ICT support	109.394	<0.001
Q0.5 vs. Q0.9	AI support in education	111.079	<0.001
Q0.1 vs. Q0.9	University ICT support	21.508	<0.001
Q0.1 vs. Q0.9	AI support in education	0.357	0.550
